# Microgravity as a Tool to Investigate Cancer Induction in Pleura Mesothelial Cells

**DOI:** 10.3390/cimb46100647

**Published:** 2024-09-27

**Authors:** Valentina Bonetto, Corinna Anais Pagano, Maurizio Sabbatini, Valeria Magnelli, Massimo Donadelli, Emilio Marengo, Maria Angela Masini

**Affiliations:** 1 Department of Science and Innovation Technology (DISIT), Università del Piemonte Orientale, 15121 Alessandria, Italy; valentina.bonetto@uniupo.it (V.B.); corinnaanais.pagano@uniupo.it (C.A.P.); valeria.magnelli@uniupo.it (V.M.); emilio.marengo@uniupo.it (E.M.); maria.masini@uniupo.it (M.A.M.); 2 Department of Neurosciences, Biomedicine and Movement Sciences (DNBM), University of Verona, 37124 Verona, Italy; massimo.donadelli@univr.it

**Keywords:** MeT-5A cells, BR95 cells, mesothelioma, focal contacts, connexin-43, NANOG, Fibulin-3

## Abstract

The present work shows that the exposure of mesothelial cells to simulated microgravity changes their cytoskeleton and adhesion proteins, leading to a cell switch from normal towards tumoral cells. Immunohistochemical and molecular data were obtained from both MeT-5A exposed to simulated microgravity and BR95 mesothelioma cell lines. Simulated microgravity was found to affect the expression of actin, vinculin, and connexin-43, altering their quantitative and spatial distribution pattern inside the cell. The analysis of the tumoral markers p27, CD44, Fibulin-3, and NANOG and the expression of genes related to cancer transformation such as NANOG, CDH-1, and Zeb-1 showed that the simulated microgravity environment led to expression patterns in MeT-5A cells similar to those observed in BR95 cells. The alteration in both quantitative expression and structural organization of the cytoskeleton and adhesion/communication proteins can thus be considered a pivotal mechanism involved in the cellular shift towards tumoral progression.

## 1. Introduction

Cell cancer transformation originates from both exogenous and intrinsic factors inducing permanent DNA alterations [1]. According to established hallmarks, transformed cells exhibit recovered or increased prolificity, upregulation of metabolism, and loss of the contact inhibition pathways [1]. These changes enable them to invade adjacent tissues and even to migrate at a certain distance, leading to their invasive/metastatic behavior [1]. A key mechanism driving cellular transformation is the alterations of cytoskeleton dynamics [2] and adhesion profile, characterized by changes in focal adhesions and their associated proteins. These transformations lead to the overcoming of contact inhibition, allowing cancer cells to hyper-proliferate and invade other tissues [3,4]. Nevertheless, the cells exhibit a resistance to alterations of their cytoskeletal architecture and adhesive interactions. When these factors are rapidly altered, the cells initiate an apoptotic pathway linked to cytoskeletal disruption [5,6] or anoikis (a programmed cell death induced upon cell detachment from the extracellular matrix, a mechanism that prevents cells’ growth or attachment to an inappropriate matrix) [7]. A potential mechanism for evading anoikis, involving complex cytoskeletal alterations and molecular adhesion profiles, is performed by the Rho-associated protein kinases (ROCKs) pathways, which play a crucial role in actin polymerization, cell migration, and morphology. Its altered expression has been observed in several tumors [4,7].

Mesothelioma is a rare and aggressive tumor primarily attributed to exogenous cytotoxic factors, such as asbestos fibers, a well-known causative factor in asbestosis, recognized in occupational [8] and in environmental exposure [9].

However, several genetic modifications are potentially responsible for mesothelioma induction, such as cyclin-dependent kinase inhibitor 2A (CDKN2A), neurofibromin 2 (NF2), large tumor suppressor 2 (LATS2), and the p53 gene (TP53) [10,11]. A characteristic feature of this tumor is the extended latency period during which pro-tumorigenic conditions persist. Chronic inflammation of mesothelial cells and the consequent reactive oxygen species (ROS) production are the main factors underlying the pathogenesis of mesothelioma. Inflammation activates a plethora of mechanisms inducing over time gene alterations leading to the malignant transformation of mesothelial cells [10].

This slow and late onset of the disease represents a significant clinical challenge for therapeutic intervention.

Gravity is a fundamental force of nature affecting the structural organization of all biological entities. Several studies on cellular systems exposed to simulated microgravity (SMG) have demonstrated that alterations in the gravitational vector disrupt cytoskeletal integrity therefore impacting on several pathways associated with cytoskeletal organization, such as those concerning the formation of adhesion complexes [12,13,14,15,16].

Given the role of cytoskeletal alterations in mesothelioma [17], studying the effects of microgravity, a condition known to disrupt cytoskeletal organization, can provide valuable insights into the transformation from normal to malignant cells.

According to the Tensegrity hypothesis, the cytoskeleton has been proposed as the structure involved in mediating the transfer of mechanical signals from the cell surface to the nucleus and consequent activation of biochemical pathways [18,19]. Microgravity has been observed to alter the tensile mechanical forces, thereby altering the physiological polymerization of the cytoskeleton and the expression of adhesion molecular complexes [18,19]. In our work, we investigated whether these dynamics can induce immortalized mesothelial cells to cancerous transformation and to what extent these changes can initiate a malignant transformation.

In the present work, we investigated the induction of cancer transformation in non-malignant mesothelioma MeT-5A cells exposed to simulated microgravity conditions using a 3D-Random Positioning Machine (3D-RPM), focusing on alterations in cytoskeletal dynamics and adhesion profiles [16]. Additionally, molecular markers and gene expression profiles associated with the acquired tumor phenotype were assessed in cells exposed to simulated microgravity. The epithelioid mesothelioma cell line BR95 was used as a cancer control to compare with the SMG-exposed Met-5A mesothelial cells.

This study aims to broaden our understanding of cancer cell biology by using the role of microgravity as a tool to achieve cytoskeletal disruption and to shed light on its contribution to early cancer transformation.

## 2. Materials and Methods

### 2.1. Cell Culture and Simulated Microgravity Exposure

MeT-5A CRL-9444 ™ cell lines, derived from mesothelium isolated from pleural fluids of non-cancerous individuals, were purchased from the American Type Culture Collection (ATCC, Manassas, VA, USA). As a very widely used cellular model of non-cancerous mesothelial cell line, the MeT-5A cell line was specifically chosen for the present study. This cell line has been immortalized and can ensure resistance and proliferation during mechanical experimental procedures that may damage other cellular lines. BR95 (epithelioid histotype) cells, derived from pleural effusion of untreated patients suffering from MPM, were cultured in Ham’s F10 (Merck, Darmstadt, Germany) culture medium supplemented with 10% fetal bovine serum (FBS, Sigma-Aldrich, Saint Louis, MO, USA) and 1% penicillin and streptomycin (PS, Sigma), at 37 °C with 5% CO_2_. The cells were kindly provided by Prof. M. Donadelli (Azienda Ospedaliera di Verona). The BR95 cell line was chosen as the malignant pleural mesothelioma cell line because of its epithelioid nature, which makes it morphologically comparable to MeT-5A.

For simulated microgravity (SMG) experiments, 5 × 10^5^ cells/mL were seeded in Nunc Slide Flask Cell Culture (Nunc™ Lab-Tek™ Flask on Slide, Thermo Fisher Scientific, Waltham, MA, USA).

Flask on Slides containing subconfluent monolayers were filled with medium to avoid the formation of air bubbles and fixed onto a Random Positioning Machine (RPM, Dutch Space, The Hague, The Netherlands) as close as possible to the center of the platform to minimize any centrifugal acceleration, and kept under continuous rotation at a speed of 56 deg/s at 37 °C for 24 h, 48 h, and 7 days. Ground control cells (1 g) were kept at 37 °C with 5% CO_2_. Controls were analyzed at the same time points previously indicated.

Floating cell clumps were observed in the cell culture supernatant of SMG-exposed cells (24 h, 48 h, and 7 days). They were collected and subsequently analyzed.

### 2.2. Immunocytochemistry (ICC) and Confocal Laser Scanning Microscopy (CLSM)

SMG cells, SMG cell clumps, frame, and ground control cells were fixed using Cy-Fix (BioOptica, Milan, Italy) fixative for 10 min at room temperature (RT) and washed three times for 5 min with PBS 1X. The cells were then permeabilized for 30 min using PBS 1X, 3% BSA, and 0.1% Triton X-100, followed by overnight incubation at 4 °C with primary antibodies.

The following primary antibodies were used: anti-vinculin (monoclonal mouse, Santa Cruz, Dallas, TX, USA; sc-73614, 1:100), anti-connexin43 (monoclonal mouse, Santa Cruz, sc-271837; 1:100), and anti-α-tubulin (monoclonal mouse, Sigma-Aldrich, T5168, 1:100). Following a PBS 1X wash, cells were incubated with the appropriate secondary antibody, FITC, or TRITC conjugated (1:150, Invitrogen Molecular Probes, Eugene, OR, USA), for one hour at RT. The same procedure was followed for negative controls as previously described. Rhodamine phalloidin (Invitrogen Molecular Probes Eugene, 1:50) was used to visualize F-actin. Cells were then washed in PBS and mounted in buffered glycerol (0.1 M, pH 9.5). Lastly, an Apotome confocal microscope with Ar/ArKr and He/Ne lasers (Leica Microsystems Heidelberg GmbH, Mannheim, Germany) was used for image acquisition. For TRITC and FITC excitation, laser lines at 543 nm and 488 nm, respectively, were used.

### 2.3. Western Blot Analysis

Samples were collected, washed in PBS 1X, and resuspended in RIPA lysis buffer (Sigma-Aldrich). A mix of protease inhibitors (Complete-Mini Protease Inhibitor Cocktail Tablets, Roche, Mannheim, Germany) and phosphatase inhibitors (PhosStop; Roche, Basel, Switzerland) were added just before use. Cellular extracts were then centrifuged at 8000× *g* for 10 min and the supernatants were collected. The protein concentration was determined with Bradford assay. Twenty-five µg of proteins for each sample were separated onto SDS polyacrylamide gels with a concentration of acrylamide specifically for the proteins of interest. The proteins were then transferred to nitrocellulose membranes (BIO-RAD, Bio-Rad Laboratories, Hercules, CA, USA). After blocking (1 h in 5% non-fat milk in TBS- 0.1% Tween20 buffer), the membranes were incubated overnight at 4 °C with the following primary anti-human antibodies: mouse anti-α-Tubulin (1:1000, Merckmillipore Burlington, MA, USA); mouse anti-β-Actin (1:1000, Cell Signaling, Danvers, MA, USA); mouse anti-CD44 (1:1000; Santa-Cruz); mouse anti-p27 (1:1000, Santa-Cruz); mouse anti-fibulina3 (1:1000, Santa-Cruz); mouse anti-vinculin (dilution 1:200; eBioscience, San Diego, CA, USA); mouse anti-connexin-43 (dilution 1:200; eBioscience); and mouse anti-NANOG (1:1000, Santa Cruz). The membranes were then incubated with the corresponding HRP-conjugated secondary antibody (1:2000; eBioscience) for 1h at RT. An ECL detection system was used to identify proteins (Amersham Biosciences, Little Chalfont, Buckinghamshire, UK).

Western blot images were acquired using a ChemiDoc™ XRS Imaging System (Bio-Rad, Hercules, CA, USA) and bands were quantified through NIH ImageJ software (Version 1.53f51, National Institutes of Health (NIH), Bethesda, MA, USA). The results represent a semi-quantitative densitometric analysis of the optical density (OD) of a specific protein signal.

Each band was evaluated as a %grey scale index × area^−4^ in pixel (OD); the optical fundus value near to the bands was evaluated as a covariate and subtracted. The OD values were normalized with the OD values of loading control represented by α-tubulin expression (mouse anti-α-tubulin, 1:1000, Merckmillipore) and expressed as relative optical density (R.O.D.). α-tubulin was chosen as the reference because, even if its polymerization is altered by microgravity, its amount into the cell was observed to be stable during the microgravity exposition in the time lapse considered in the present research [11,15]. Data of the α-tubulin western blot were evaluated as densitometric areas in the pixel, normalized for background.

### 2.4. RT-qPCR

The total RNA was extracted from MeT-5A and BR95 cells cultured in different experimental conditions using TRIzol™ Reagent (Thermo Fisher Scientific). The reverse transcription of 1 μg of RNA was performed using first-strand cDNA synthesis. RT-qPCR was performed through SYBR-Green detection chemistry with GoTaq qPCR Master Mix (Promega, Madison, WI, USA). PCR reaction was carried out with QuantStudio 3 Real-Time PCR System (Thermo Fisher Scientific, USA). The thermal protocol consisted of 95 °C for 10 min, 40 cycles at 95 °C for 15 s, 60 °C for 1 min, 95 °C for 15 s, and 60 °C for 15 s. Relative gene expression was calculated according to the 2^−ΔΔCt^ method. In addition, 18S gene expression was used as an endogenous control to normalize mRNA expression.

Primers are listed in Table 1.

### 2.5. Statistical Analysis

The data are reported as mean ± SD of three repeated experiments. The statistical analysis was performed using a two-way ANOVA to investigate statistical differences between CLUMP vs. Adherent cells (the first variance source) and between different times of exposition to microgravity (the second variance source). Statistical differences among experimental groups f analyzed were then assessed by one-way ANOVA followed by a post hoc Bonferroni test (GraphPad Prism v.8 GraphPad Software). The normality of the data distribution was assured by the Kolmogorov–Smirnoff test. Values of *p* < 0.05 were considered significant.

## 3. Results

Cell cultures under the normogravity condition (1 g) exhibited a predominantly flattened and adherent morphology with a minor population of rounded cells. Extensive analysis of several cellular parameters (detailed analysis below) revealed no significant changes over the experimental timeframe (24 h, 48 h, and 7 days). Consequently, the data were collectively presented as the ground control group.

**Figure 1:**
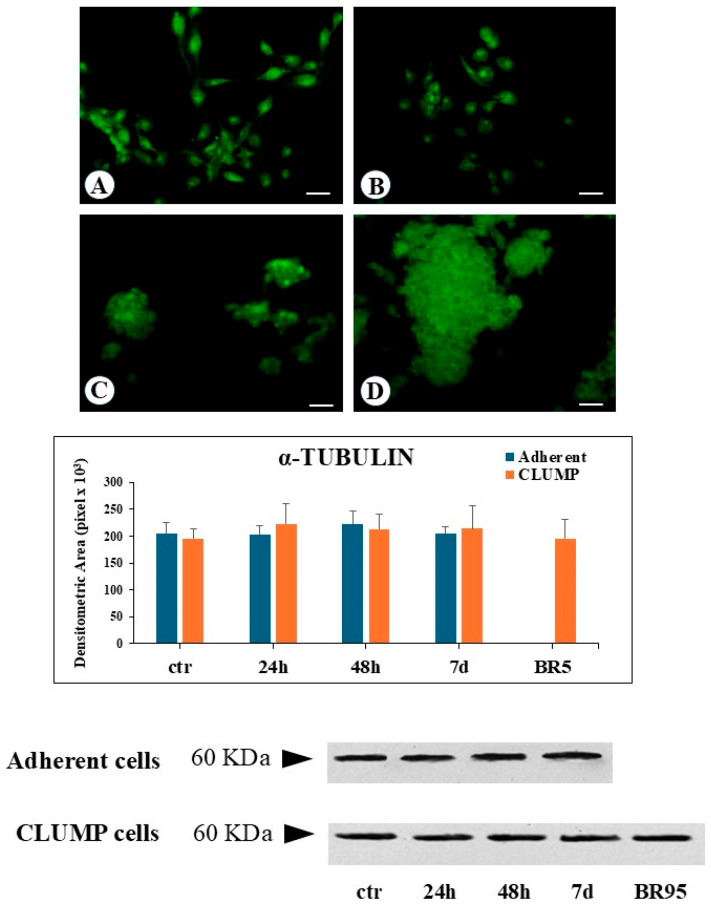
Microphotograph panel illustrating the immunofluorescence localization of α-Tubulin in MeT5A cells (**A**,**B**) in their agglomerate (CLUMP) disposition (**C**) and BR95 cells (**D**), following normogravity (**A**) or microgravity at 48 h (**B**,**C**). The quantitative expression of several proteins analyzed is shown in the bar-graph and western blot panel. Calibration bar = 20 µm. No statistical difference detected. N = 3.

Simulated microgravity exposure led to the appearance of more flattened adherent cells. Starting from the 24 h time point, rounded, detached cells were observed to form clusters, identified as a floating group of five or more collecting cells (CLUMP cells, Figure 1B,C). The number of these cell clusters progressively increased with prolonged simulated microgravity exposure.

The two-way ANOVA (TWA) test validated the independence of the experimental group’s values for actin, vinculin, connexin-43, CD44, and fibulin-3, and confirmed the significance analyzed by one-way ANOVA as detailed below. While for p27 and NANOG, the TWA analysis confirmed a similar variability within the adherent and CLUMP experimental groups. For tubulin, the TWA analysis confirmed the absence of significant differences between the different experimental groups analyzed.

### 3.1. Cytoskeleton

The modifications of the cytoskeleton have been evaluated by analyzing microtubule and actin changes induced by SMG.

Microtubules are formed by polymerization of tubulin dimers, resulting in 13 linear protofilaments assembled around a hollow core. Each dimer is composed of α-tubulin and β-tubulin proteins. In the present research, we predominantly focused our attention on modifications of α-tubulin. Immunofluorescence analysis indicates that SMG induced a change in the microtubular net, which became fully disarranged and fragmented with a worst progression over time, both in adherent and in CLUMP cells. This phenotype was very similar to disassembled and fragmented microtubules observed in the BR95 cell line (Figure 1). Western blot analysis revealed a consistent α-tubulin protein expression level in both adherent and CLUMP cells across all SMG exposure durations. Similarly, no variation in α-tubulin abundance was observed in the BR95 cell line (Figure 1).

**Figure 2:**
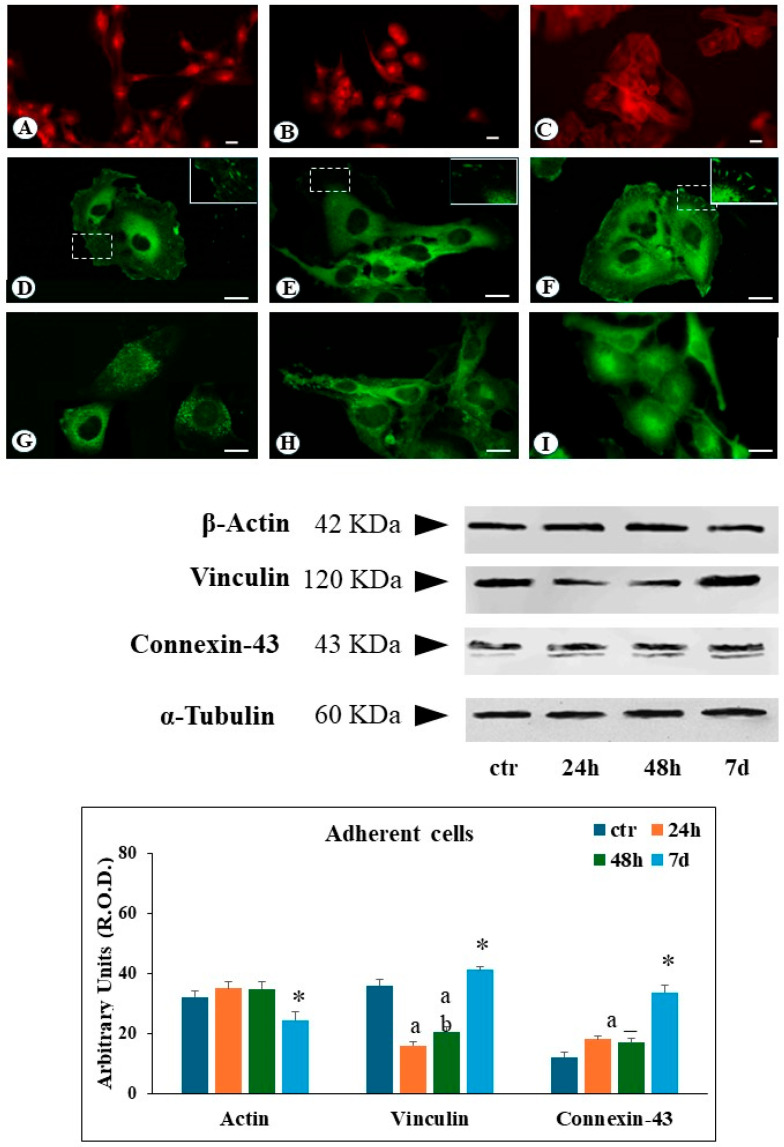
Microphotograph panel illustrating the immunofluorescence localization of Actin (**A**–**C**), Vinculin (**D**–**F**), and connexin-43 (**G**–**I**) in adherent MeT5A cells, following normogravity (**A**,**D**,**G**) or microgravity at 48 h (**B**,**E**,**H**) and 7 days (**C**,**F**,**I**). In (**D**–**F**), the frame shows the observed focal contacts in high detail. The quantitative expression of several proteins is shown in the bar-graph and western blot panel. Calibration bar = 20 µm. a = *p* < 0.05 vs. ctr; b = *p* < 0.05 vs. 24 h; * = *p* < 0.05 vs. other experimental groups. N = 3.

Actin filaments constitute a critical component of the cytoskeleton, primarily responsible for cellular motility and regulating cell–extracellular matrix interactions. Immunofluorescence analysis of ground control cells revealed well-defined, organized actin filaments distributed throughout the cytosol, with additional actin fibers located in close proximity to the plasma membrane (Figure 2A).

Following simulated microgravity exposure, cytosolic actin filaments became increasingly difficult to recognize after 24 h, with the appearance of an evident diffuse cytoplasmic immunofluorescence pattern. At 48 h and 7 days, a significant reduction of actin filaments was observed, compared to ground control, with the remaining actin structures localized to the cell periphery (Figure 2B,C). Western Blot analysis of adherent cells revealed no significant changes in the total amount of actin protein levels following up to 48 h of SMG exposure. However, a decrease in actin expression was detected after 7 days of SMG condition (Figure 2).

**Figure 3:**
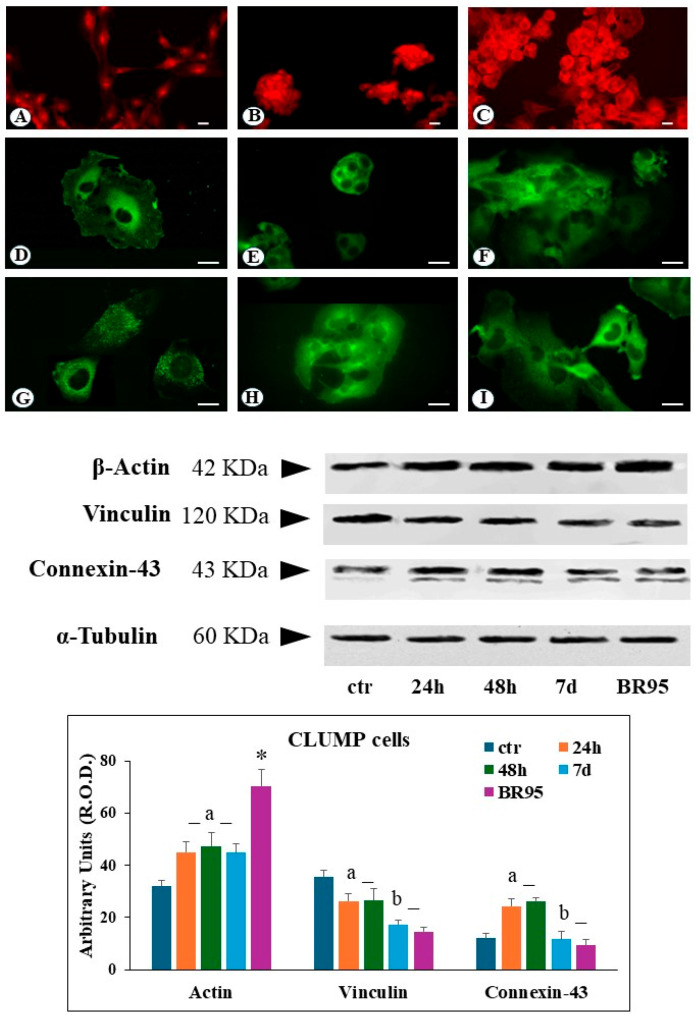
Microphotograph panel illustrating the immunofluorescence localization of Actin (**A**–**C**), Vinculin (**D**–**F**), and connexin-43 (**G**–**I**) in adherent MeT5A cells (**A**,**D**,**G**) in their agglomerate (CLUMP) disposition (**B**,**E**,**H**) and adherent BR95 cells (**C**,**F**,**I**), following normogravity (**A**,**D**,**G**) or microgravity at 7 days (**B**,**E**,**H**). The quantitative expression of several proteins analyzed is shown in the bar-graph and western blot panel. Calibration bar = 20 µm. a = *p* < 0.05 vs. ctr; b = *p* < 0.05 vs. 24 h; * = *p* < 0.05 vs. other experimental groups. N = 3.

In CLUMP cells, actin filaments appeared shorter, fragmented, and reduced, localized at the perinuclear site following 24 h to 7 days of SMG exposure. A similar organization was observed in the BR95 cell line (Figure 3B,C). Western blot analysis of CLUMP cells revealed a significant increase in total actin protein levels compared to the ground control cells following 24 h to 7 days of SMG exposure. A somewhat higher expression of total actin content was detected in the BR95 cell line compared to both CLUMP and ground control (Figure 3).

### 3.2. Cell-to-ECM Interactions

Cell–extracellular matrix (ECM) interactions are mediated by cell surface receptors and specific protein complexes that transduce mechanical signals into intracellular pathways. Vinculin is a membrane-cytoskeleton protein involved in the focal adhesion plaque’s formation and stability. The reduction of vinculin expression in focal contacts has been associated with metastatic behavior of cancer cells [14].

Ground control cells reveal a regular distribution of focal contacts along the cell periphery, with additional perinuclear immunofluorescence staining (Figure 2D). In contrast, after 24 and 48 h of SMG exposure, adherent cells showed a substantial reduction of the number of focal contacts, and vinculin immunoreactivity was widely detected in the cytosol (Figure 2E). Following 7 days of simulated microgravity, adherent cells showed a recovery in the number of vinculin-positive focal contacts, alongside a strong cytosolic vinculin signal (Figure 2F). Western blot analysis on adherent cells revealed the reduction of the total amount of vinculin protein levels during the 24–48 h exposure period, followed by their increase after 7 days (Figure 2). In contrast to adherent cells, CLUMP cells exhibited a predominant cytosolic localization of vinculin, consistent with the lack of adherent interactions (Figure 3E). A similar pattern was observed in the BR95 cell line (Figure 3F). In the CLUMP cells, the western blot analysis showed a decrease in the total amount of vinculin during the 24–48 h interval, with a further reduction observed after 7 days of exposure; the BR95 cell line exhibited vinculin levels comparable to those of MeT-5A cells following 7 days of SMG exposure (Figure 3).

Connexin-43 is a major component of gap junction proteins mediating cell-to-external environment communication. Connexin-43 has a paramount role in ions and small molecule exchange across the cellular membrane [20]. In ground control cells, connexin-43 is clearly organized around the cells, suggesting an effective communication of the cell with the external environment (Figure 2G). Cells exposed to SMG for 24 to 48 h had a strong reduction of the immunofluorescence signal along the cellular boundaries, whilst a pronounced increase in cytosolic immunofluorescence flattened adhering cells (Figure 2H). Seven days of SMG exposure resulted in an increased perinuclear immunofluorescence signal in adherent cells (Figure 2I).

In the case of adherent cells, western blot analysis showed an increase in connexin-43 amount following 24 and 48 h of SMG and a further rise after 7 days (Figure 2).

CLUMP cells exhibited an increase in connexin-43 expression with major localization in the perinuclear zone at 24–48 h of SMG exposure (Figure 3H). Instead, a decrease in connexin-43 expression was detected following 7 days; a similar connexin-43 expression was detected in the BR95 cell line (Figure 3H,I). Western blot analysis in CLUMP cells showed an increase in connexin-43 between 24 and 48 h, followed by a substantial downregulation after seven days, similar to the pattern detected in the BR95 cell line (Figure 3).

### 3.3. Staminal Phenotype Analysis

The acquisition of staminal phenotype was evaluated in adherent cells, in CLUMP cells, and in the BR95 cell line by western blot analysis using the expression of NANOG protein and CD44 [21,22] as markers.

**Figure 4:**
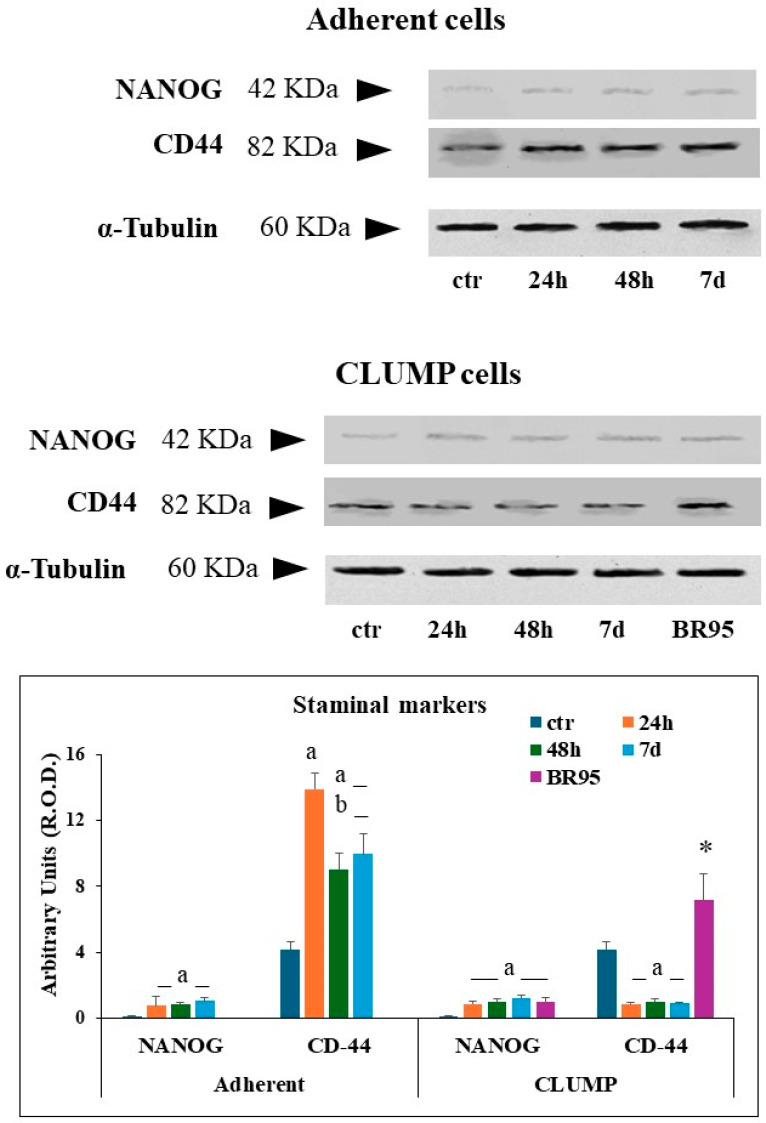
Panel illustrating the expression of several proteins analyzed in adherent MeT5A cells. The quantitative expression of several proteins analyzed is shown in the bar-graph. a = *p* < 0.05 vs. ctr; b = *p* < 0.05 vs. 24 h; * = *p* < 0.05 vs. other experimental groups. N = 3.

The expression of NANOG was very low in Met-5A ground control cells. NANOG expression increased within 24 h of SMG and remained stable up to 7 days. CD44 expression was detected in Met-5A ground control cells, with an increasing trend within the first 24 h, followed by a partial reduction at 48 h and 7 days (Figure 4).

In CLUMP cells, NANOG expression became evident at 24 h as well, reaching a steady-state expression maintained up to 7 days. BR95 cells showed results similar to those observed in MeT5A cells at 7 days. Instead, CLUMP cells exposed to SMG showed a reduced level of CD44 over time, whereas BR95 cells maintained a higher CD44 expression compared to that of Met-5A ground control cells (Figure 4).

### 3.4. Tumor Markers

Fibulin-3 and the onco-suppressor p27 were evaluated to investigate the occurrence of tumor markers in the MeT5A adherent and CLUMP cells and BR95 cell line.

Fibulin-3 is a secreted ECM glycoprotein representing an early detection prognostic biomarker for malignant pleural mesothelioma (MPM) [23]. Whereas p27 is a cellular cycle inhibitor and onco-suppressor, which is considered a biomarker for cancer transformation upon its downregulation [24].

**Figure 5:**
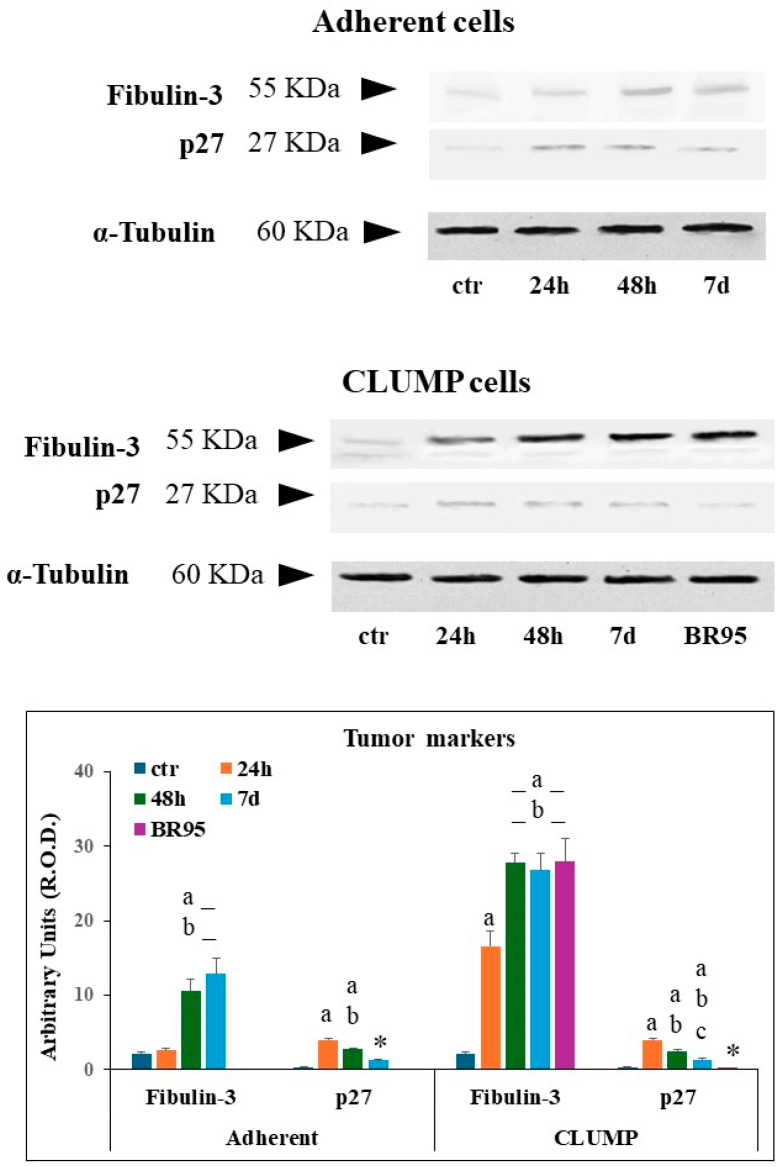
Panel illustrating the expression of several proteins analyzed in MeT5A cells in their (CLUMP) disposition. The quantitative expression of several proteins analyzed is shown in the bar-graph. a = *p* < 0.05 vs. ctr; b = *p* < 0.05 vs. 24 h; c = *p* < 0.05 vs. 48 h; * = *p* < 0.05 vs. other experimental groups. N = 3.

Western blot analysis of Fibulin-3 showed a mild signal in ground control MeT-5A cells and in adherent cells exposed to SMG for 24 h, while a marked increased expression was detectable at 48 h and 7 days (Figure 5). Instead, Met-5A ground control cells exhibited minimal p27 expression. Adherent cells displayed a marked increase in p27 within the first 24 h of SMG exposure, followed by a decrease; however, p27 levels remained compared to the ground control group (Figure 5).

In CLUMP cells, the expression of fibulin-3 strongly increased starting from 24 h and reached a high steady-state level at 48 h and 7 days. As expected, a very high expression level was detected in BR95 cancer cells (Figure 5). The p27 expression increased in CLUMP cells following SMG application for 24 h, then gradually declined at 48 h and 7 days. As expected, the expression of p27 was sparse in the BR-95 tumoral cell line (Figure 5).

### 3.5. Epithelial–Mesenchymal Transition in CLUMP Cells

The above morphological data suggest a shift of adherent cells to a floating phenotype (CLUMP) indicating an underlying cancer transformation process. We focused on the expression of possible stemness markers to validate the transformation hypothesis. For this purpose, we considered NANOG, a transcription factor that controls self-renewal and pluripotency of embryonic stem cells; CDH-1, involved in the regulation of cell–cell adhesions, mobility, and proliferation of epithelial cells; and Zeb-1, a zinc-finger transcription factor inducing epithelial–mesenchymal transition [25,26]. The *NANOG*, *Zeb-1*, and *CHD-1* gene expression has been analyzed by RT-qPCR and the results were compared with the BR95 cell line.

**Figure 6:**
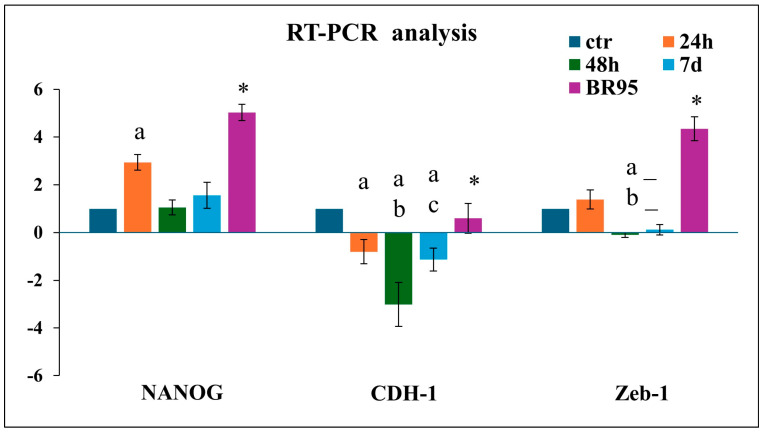
Bar-graph illustrating the expression of several gene analyzed in MeT5A cells in their (CLUMP) disposition, following normogravity or microgravity application and in adherent BR95 cells. a = *p* < 0.05 vs. ctr; b = *p* < 0.05 vs. 24 h; c = *p* < 0.05 vs. 48 h; * = *p* < 0.05 vs. other experimental groups. N = 3.

MET-5A CLUMP cells exposed to 24 h of SMG revealed a strong increase in *NANOG* expression, followed by a decrease at 48 h and 7 days compared to ground control cells. A decrease in *CDH-1* was detected after 48 h. Zeb-1 showed a modest increase at 24 h followed by a reduction at 48 h and 7 days compared to ground control (Figure 6). Instead, a high expression of *NANOG* and *Zeb-1* and an expression of *CDH-1*, similar to that detected in ground control cells characterized BR95 cancer cells, well identify their mesenchymal character (Figure 6).

## 4. Discussion

The cytoskeleton provides structure for the eukaryotic cells and drives the adhesion interactions with the extracellular substrate. Disruption of the cell–extracellular matrix (ECM) interactions triggers anoikis, a form of substrate-dependent cell death [27]. This process is mediated by cytoskeletal and cellular adhesion signals activating apoptotic pathways [5]. One of cancer cells’ hallmarks is the activation of invasion and metastasis programs, enabling colonization of distal anatomical sites. The acquisition of stem-like properties, motility, and sustained proliferative signaling drive the invasive and metastatic potential of tumor cells [5,8].

The cytoskeleton is a primary cellular sensor of mechanical stimuli, exhibiting significant alterations in response to changes in gravity [13]. Several authors report a strong correlation between cell mechanical properties and cancer leading to changes in cell behavior and tumor microenvironment [28,29].

Some MeT-5A cells exposed to simulated SMG do not adhere to the flask substrate, switching to floating clusters with different shapes and sizes. This phenotypic transition occurs within the first 24 h of SMG exposure. The initial 48 h of SMG are critical to let the cytoskeleton assume new characteristics, leading to the loss of adhesive properties and communication complexes responsible for the floating state. Similar results have been proposed for MCF-7 cells [19,30]. This behavior is associated with a cytoskeleton reorganization, mainly due to alterations in the network of actin microfilaments and microtubules. Further, we detected significant alterations in the distribution and expression of proteins related to cell adhesion complexes such as vinculin, fibulin-3, CD44, and cellular communication proteins, connexin-43. A fundamental aspect to evaluate is that MeT-5A cells in a floating state share common characteristics found in mesothelioma cells. In contrast, adherent cells show enhanced adhesion-promoting factors.

The redistribution of adhesion and communication proteins such as vinculin and connexin-43 reflects pivotal intracytoplasmic changes which underline possible alternative pathways for floating cells. Vinculin molecules are distributed between a cytoplasmic pool and adhesive plaques, with the latter contributing significantly to cellular stability [31]. It has been shown that the conformation of cytoplasmic vinculin is an inactive form with respect to its actin-binding potential [32]. In our model, the vinculin cytoplasmic increase and the corresponding reduction in the membrane content is suggestive of a loss of relationship with the surrounding environment, driving towards a form of independence which supports the evolution of the cell towards tumor characteristics [33]. Similarly, the shift of connexin-43 from its membrane localization to form a cytoplasmic pool could be due to the disassembly of gap junctions connected with the activation of phosphorylation pathways [34] induced by SMG exposure. This switch would increase the proliferative rate and metabolic capabilities of the cells [16,35,36].

Interestingly, in the adherent cells the expression of cytoplasmic connexin-43 continues to increase over longer periods of SMG exposure, probably following the activation of specific surviving pathways [36]. Indeed, cytoplasmic connexin-43 activity has been associated with upregulation of p27 [37]. p27, an inhibitor of cyclin-dependent protein kinases (CDKs) that control cell cycle progression, is recognized as a tumor suppressor. Downregulation has been consistently observed in a wide range of tumors [38,39,40], including mesothelioma, where p27 downregulation is a molecular prognostic marker [24,41]. In free-floating cells, connexin-43 was reduced both in the cytoplasmic pool and in the membrane, exhibiting similar levels to mesothelioma cells.

The dislocation of connexin-43 would therefore be considered a trustable indicator of early cancerous transformation of floating cells expressing low levels of p27. In contrast, adherent cells reinforce adhesion molecular structures, activating pathways associated with tumor suppression, mainly through p27 upregulation. A similar situation was revealed from the changes in CD44 expression levels.

CD44 is a transmembrane glycoprotein binding hyaluronic acid (HA) of the extracellular matrix and intracellular cytoskeletal elements such as actin. Its phosphorylation can directly trigger intracellular signaling, promoting cell proliferation, motility, and invasion and, thus, supporting metastatic invasion [22]. The interaction of CD44 with its ligand HA has revealed an important role in modulating cell proliferation and invasiveness in mesothelioma. An increase in CD44 expression was detected in cells involved in the early stages of mesothelioma (tumor initiating cells, TICs) [42]. In consideration of this, CD44 has been evaluated as potential biomarker for mesothelioma [43]. An increase in the synthesis of the membrane protein CD44 correlates well with the cytoskeletal rearrangement, supporting the migration process of the cells which thus change from adhesion to suspension condition. The relationship between CD44 and actin in SMG is confirmed by the observed trend of these two proteins. Cells exposed to SMG for 24 h displayed an increase in the expression of both actin and CD44, corresponding to the peak of the cytoskeletal remodeling. A significant decrease in their expression levels was observed after 48 h of exposure in cells undergoing the adherent-to-floating phenotypic transition, concurrent with cytoskeletal stabilization.

In order to have indications concerning the tumor transformation, we analyzed the expression of fibulin-3, a secreted extracellular matrix glycoprotein which interacts with regulatory molecules of the extracellular matrix, anchoring them to the basement membranes [44]. High levels of fibulin-3 have been found in the pleural fluid of patients with MPM, and they correlate with poorer survival [45]. Fibulin-3 blood levels can distinguish malignant mesothelioma effusions from other malignant and benign effusions and can, thus, be seen as a biomarker of the malignant form [23]. Overexpression of fibulin-3 activates Pi3K/Akt/MAPK pathways, triggering cells to proliferate and acquire metastatic properties [46]. The high expression of fibulin-3 following long exposure to SMG in adherent cells and in detached cells strongly support the hypothesis of the cancer-inductive effect of SMG on mesothelial cells.

A typical phenomenon of tumor cells’ transformation is the epithelial–mesenchymal transition (EMT), a process in which epithelial cells acquire motility and invasive characteristics of mesenchymal cells. Although EMT can be seen as a process connected to organism development, it is strictly responsible for metastatic diffusion of cancer cells, supporting tumor progression, invasiveness, and therapy resistance. The acquisition of the mesenchymal phenotype represents a pivotal step in cancer cell transformation [26,47]. EMT has a significant role in the evolution of malignant mesothelioma [48], and many EMT genes have been identified in malignant mesothelioma [49].

The *NANOG* gene induces the expression of a transcription factor in staminal cells, and it is considered a fundamental factor to maintain pluripotency [50]. *NANOG* is downregulated during cellular differentiation but is re-expressed in many cancer cells, with higher levels observed in more aggressive and metastatic tumors [51]. *NANOG* is currently considered an oncogene [25,52] involved in the main processes of cancer cell proliferation, but also in EMT and metastatic evolution [21]. SMG exposure increased the expression of the *NANOG* gene in adherent and floating cells, driving the transformation in tumor cells.

Another event observed in cancer cells’ transformation consists of E-cadherin decrease. E-cadherin, encoded by the *CDH-1* gene, is an important cell adhesion molecule which mediates the cell-to-cell contacts, being the main molecule in adherent junctions that connect neighboring epithelial cells. E-cadherin reduction is a hallmark step in the acquisition of a mesenchymal phenotype, which normally occurs during embryogenesis [53]. Unfortunately, in cancer progression, E-cadherin downregulation favors progression and invasiveness [54]. The sharp reduction of *CDH-1* following SMG exposure supports the occurrence of pathways culminating in cancerous transformation of the cells.

*Zeb-1* belongs to the Zinc-finger Homeobox (ZFH) family of repressors. It is a transcription factor which promotes tissue invasion and metastasis acting as a repressor of *CDH-1* expression [55]. SMG did not change its expression in our cellular model.

While the proposed conclusion suggests effective biochemical pathways, further experimental evidence is needed to firmly establish their involvement. Several steps in the process remain speculative and require additional research to validate. In summary, while the experimental data support the involvement of fibulin-3 in the regulation of connexin-43 redistribution and epithelial–mesenchymal transition (EMT), further research is needed to definitively confirm the specific mechanisms involved, such as the activation of the Pi3K/Akt/MAPK pathways and the induction of EMT-related genes. Connexin-43 acts as an inhibitor of p27, a recognized tumor suppressor factor. Pi3K/Akt/MAPK pathways and connexin-43 may in turn involve alterations of the cytoskeleton and adhesion complexes, ultimately favoring cancer transformation of cells.

This study aims to investigate the critical role of the cytoskeleton and associated pathways in the early stages of cancer development by utilizing microgravity conditions. By analyzing these factors, we hope to identify potential biomarkers that could improve cancer diagnosis and prognosis. Furthermore, our findings support the established role of CD44 and p27 as valuable biomarkers for cancer diagnosis and prognosis. Additionally, our study highlights the potential significance of connexin-43 as a promising biomarker for cancer progression.

### Limitations and Perspectives

The use of the MeT-5A cell line as the experimental group and BR95 as the malignant counterpart has the limitation of potentially restricting our findings to these specific cell types. While our data may be applicable to other similar cells, further validation with a broader range of cell lines is necessary to generalize the results. Additionally, the flattened morphology of MeT-5A cells and the epithelioid nature of BR95 mesothelioma cells primarily focus our investigation on epithelioid-type mesothelioma.

Given that the gravitational force acts equally on all parts of a cell or an animal body, we anticipate that isolated cells or cells within an organism might exhibit similar responses to simulated microgravity. Despite the uniform effect of gravity, intracellular factors like cell–cell and cell–extracellular matrix interactions, vascularization, and stimulating factors can significantly influence whether cells undergo transformation within the animal body [1]. Given the limitations of in vitro studies, the current research would benefit from in vivo experiments to better understand the observed cellular transformations. However, identifying the cellular structures directly affected by simulated microgravity in isolated cells represents a first investigative step [5,8]. In the present research, we found that simulated microgravity affects those cell structures recognized as largely involved in the first steps of cancerous cell transformation, supporting the validity of the present research. The following phase of this study will be dedicated to investigating the in vivo behavior of MeT-5A cells after undergoing simulated microgravity protocols.

Based on the structural and metabolic alterations acquired by MeT-5A cells after simulated microgravity treatment, it is hypothesized that these cells will exhibit a proliferative and invasive phenotype when implanted in vivo. It will also be valuable to examine the interaction between the immune system and the inoculated cells, comparing nude mice and mice with intact immune systems.

Although our work is based on simulated microgravity by the use of laboratory RPM equipment, this work is in agreement with previous results obtained by other authors such as Vassy and coll. [18] who report that, following weightless experiments during a space flight, all cytoskeleton components are widely disturbed by the alteration of gravity vector, in their structural and dynamic properties.

Nassef and coll. [19] have analyzed cytoskeletal components such as actin and tubulin during parabolic flight, using a novel approach of visual microscopic investigation, a spinning-disc Fluorescence Microscopy Analysis System (FLUMIAS), for a direct live-cell imaging over time of cytoskeletal change. These authors confirmed, using MCF-7 cells, that the alteration of gravity vector induces large alterations in the disposition of cytoskeletal elements and changes in the gene expression patterns. Similarly, the induction of detaching cells following grouping in floating masses was observed.

However, the RPM may induce limited shear forces due to the effect of fluid movements, and centrifugal acceleration. These variables have been previously evaluated under the same condition adopted in the present study, and the absence of any influence on the tested parameters was detected [16]. The effect of centrifugal acceleration has been corrected by fixing the flasks as close as possible to the center of the RPM platform.

It is known that one typical hallmark of cancer cells is their ability to reprogram metabolic pathways [56] in order to optimize energy production to support proliferation activities, especially during the metastatic phase [57]. A broader investigation should be conducted in simulated microgravity to find out possible alterations of the energy metabolic pathways, to highlight the inductive effect of microgravity on cells’ cancerous transformation.

## 5. Conclusions

In conclusion, the application of SMG on MeT-5A cells causes alterations in the adhesion and cytoskeletal stability factors, providing the acquisition of stemness characteristics and markers specifically associated with tumor transformation. We speculate that the SMG condition, which does not allow for normal cell-to-cell and cell-to-external environments, forces cells to adapt and establish a new equilibrium for survival. Further, the cellular transformation of MeT-5A diverges into two distinct paths: one involving enhanced adhesion, and another characterized by loss of adhesion and acquisition of tumor-like properties.

Our work did not consider the exploration of molecular pathways beyond the activation of several factors investigated. However, fibulin-3 is known to act via Pi3K/Akt/MAPK pathways which represent one of the most widespread and active pathways in cells [46], while ROCKs pathways play a crucial role in cytoskeletal polymerization becoming important in cell migration [4,7]. These two pathways are expected to be mainly involved in mediating the alteration of factors, gene expression and cytoskeleton polymerization, as observed in the present work. As previously indicated, the mesothelial cells inflammation has been recognized as having a key role through the secretion of cytokines and stimulating factors, which may cause the external stimuli to activate the cellular pathways [10]. ROS production itself has been recognized as being able to activate cellular pathways favoring cell transformation [10].

The reported observations emphasize the hypothesis under which the cell adhesion factors, and the cytoskeletal organization, have a paramount role in determining the physiological behavior of cells. The progressive cellular transformation in cancer onset is strictly correlated to the alterations of both these factors.

In addition to the use of microgravity as tool to investigate the factors involved in cancer induction, microgravity may also assume an evaluable opportunity for a therapeutic option in mesothelioma. While MeT-5A cells are an immortalized cell line and may not perfectly replicate primary mesothelial cells, their divergent response to microgravity—with some cells exhibiting strong substrate adhesion and others adopting a free-floating disposition—is a noteworthy observation. This double behavior paves the way for a more in-depth analysis to evaluate the potential role of microgravity as a factor for inducing cell transformation, becoming an interesting warning in the era where long-term spatial missions are being discussed. While microgravity is primarily associated with promoting cell transformation, some studies have suggested that it may also induce regression in certain tumor cell lines [16]. And a short exposure to a microgravity environment could be tested to analyze the potential help in improving therapeutic options against mesothelioma.

## Figures and Tables

**Table 1:** Sequences of primers used for qPCR analysis.

Genes	Forward Primer Sequence	Reverse Primer Sequence
CDH1	5′-GAC ACC AAC GAT AAT CCT CCGA-3′	5′-GGC ACC TGA CCC TTG TAC GT-3′
ZEB1	5′-GTT ACC AGG GAG GAG CAG TGAAA-3′	5′-GAC AGC AGT GTC TTG TTG TTG TAG AAA-3′
NANOG	5′-AGT CCC AAA GGC AAA CAA CCC ACT TC-3′	5′-TGC TGG AGG CTG AGG TAT TTC TGT CTC-3′
18S	5′-ACT TTC GAT GGT AGT CGC CGT-3′	5′-CCT TGG ATG TGG TAG CCG TTT-3′

## Data Availability

Our study is a morphological and observational study based on the distribution of specific proteins. The minimal data set of the image collection is shown in the article. Further raw data are available in Mendeley Data Repository as “Sabbatini, Maurizio; Masini, Maria Angela (2024), “Microgravity as Tool to Investigate Cancer Induction in Pleura Mesothelial cells”, Mendeley Data, V1, doi:10.17632/3z7yxc6stb.1”.
